# Comparison of Osteopathic (DO) and Allopathic (MD) Candidates Matching Into Selected Surgical Subspecialties

**DOI:** 10.7759/cureus.40566

**Published:** 2023-06-17

**Authors:** James Brazdzionis, Paras Savla, Rachel Oppenheim, Grace J Kim, Kristen Conrad-Schnetz, Bracken Burns, Alexandra Beier, David A Connett, Dan E Miulli

**Affiliations:** 1 Neurosurgery, Riverside University Health System Medical Center, Moreno Valley, USA; 2 General Surgery, Cleveland Clinic South Pointe Hospital, Warrensville Heights, USA; 3 Surgery, East Tennessee State University, Johnson City, USA; 4 Pediatric Neurological Surgery, University of Florida College of Medicine - Jacksonville, Jacksonville, USA; 5 Family Medicine, Western University of Health Sciences, Pomona, USA

**Keywords:** general surgery, neurosurgery, surgical subspecialties, do, md, allopathic, surgical, match rate, osteopathic, match trends

## Abstract

Context

Medical students and graduates apply for post-graduate year-one positions every year through the Single Accreditation System (SAS) National Residency Match Program (NRMP). New opportunities have arisen for osteopathic graduates through the transition to a single match. There is a paucity of information evaluating the effects of this single match on osteopathic (DO) and allopathic (MD) candidates in relation to match rates in competitive surgical sub-specialties such as neurosurgery, thoracic surgery, vascular surgery, otolaryngology (ENT), plastic surgery, orthopedic surgery, and general surgery.

Objectives

This paper utilizes published data to accomplish three tasks. Firstly, it investigates the effects of the SAS on DO and MD match rates in surgical subspecialties of neurosurgery, thoracic surgery, vascular surgery, ENT, plastic surgery, orthopedic surgery, and general surgery. Secondly, it investigates whether program director credentials and impressions correlate with the match rates of DO or MD candidates in each of these specialties. Finally, it discusses solutions for addressing ways to improve match outcomes for all candidates.

Methods

Previously published NRMP, National Matching Services, and Accreditation Council for Graduate Medical Education websites were queried for the number of DO and MD senior applicants for each position, match success rates, program director impressions, and program director credentials for the years 2018-2023. Match success rates were defined as a ratio of the number of candidates that applied to the number who successfully matched. Data were analyzed using descriptive statistics, chi-squared testing, student t-tests, and linear regression where appropriate. A p-value of less than 0.05 was considered significant.

Results

From 2020-2023, an increasing proportion of DO residents applied for the selected surgical subspecialties, increasing from 599 applicants in 2020 to 743 candidates in 2023. Overall match rates for DOs remain significantly lower than MD match rates for each of these specialties as well as overall (p-values all <0.05) with summative match rates of 52.89% for DOs compared to 73.61% for MDs in 2023 for the selected surgical subspecialties. From 2020 to 2023 match rates were 30.88% for DOs compared to 74.82% for MDs in neurosurgery, 16.67% versus 46.45% (DO vs MD) in thoracic surgery, 4.17% vs 68.84% (DO vs MD) in plastic surgery, 57.62% vs 73.18% (DO vs MD) in general surgery, 23.21% vs 74.18% (DO vs MD) in vascular surgery, 53.10% vs 72.57% (DO vs MD) for ENT, and 56.92% vs 72.51% (DO vs MD) for orthopedics. There was a statistically significant correlation between the proportion of DO program directors with the rate of DOs matching in the associated specialty (p=0.012).

Conclusion

There were significantly lower rates for DO candidates compared to MD candidates matching into selected surgical subspecialties of neurosurgery, thoracic surgery, vascular surgery, ENT, plastic surgery, orthopedic surgery, and general surgery. This may be addressed through increasing advocacy at local and national levels, improving mentorship, increasing DO medical student exposure to surgical subspecialties, and ensuring increasing selected surgical subspecialty involvement in teaching these diverse DO applicants in order to strengthen medicine and continue to address predicted growing physician shortages.

## Introduction

Each year medical students and graduates apply for open post-graduate year one (PGY-1) residency positions through the National Residency Matching Program (NRMP) process, American Urological Association, or the San Francisco Match. Until 2019, osteopathic medical students could also participate in the American Osteopathic Association (AOA) national match process through the National Matching Services (NMS).

Beginning in July 2015, the AOA and the Accreditation Council for Graduate Medical Education (ACGME) began a transition to a single accreditation system (SAS), combining the AOA and NRMP match programs. Between 2015 and 2020, AOA programs applied for accreditation to the ACGME. If granted ACGME accreditation, these programs would be allowed to take residents through the NRMP match. Of the approximate, 1,018 AOA programs initially accredited by the AOA, 632 applied for accreditation. In January 2018, 171 of those programs were in the pre-accreditation phase, 161 were given the status of continued pre-accreditation, 287 were considered initially accredited by the ACGME, and seven earned continued accreditation. Within this subset, AOA surgical subspecialty residencies initially appeared to have the most difficult time making the transition to ACGME accreditation. When evaluating the specialties of surgery, orthopedic surgery, neurosurgery, otolaryngology (ENT), vascular surgery, thoracic surgery, and plastic surgery, there were 134 AOA programs initially accredited by the AOA. Of these, in January 2018, only 28 had been granted the status of initial accreditation by the ACGME. By May 2020, programs had largely made the transition to the SAS or had withdrawn and were no longer taking residents. In January 2018, of the 134 AOA specialty programs, four of 10 neurosurgery programs, 40 of 42 orthopedic programs, 13 of 15 otolaryngology programs, three of three plastic surgery programs, 48 of 56 surgery programs, and one of eight vascular surgery programs for a total of 109, had made the transition to either initial or continued ACGME accreditation. The remaining AOA programs that had not made the transition to ACGME accreditation were no longer allowed to accept new residents and were allowed to complete the training of the residents remaining in their programs under AOA accreditation with planned closure upon graduation of the last remaining resident.

Once granted ACGME accreditation, these former AOA programs along with the previous ACGME programs, all participated in a singular NRMP match process with 2020 being the first year all programs were required to have made the transition to ACGME accreditation. This merger has opened doors for osteopathic medical students and residents at former AOA programs with the prospect of increased inclusion in additional residency programs. With the SAS process, and now multiple years of data, it is becoming increasingly prudent to evaluate overall match rates amongst medical students from osteopathic (DO) and allopathic (MD) programs to ensure there is equity in training opportunities for the next generation of physicians and surgeons.

From 2018 to 2020 there was an increase in the number of positions available due to the transition of previous AOA residency positions as well as de novo positions created. All MD senior applicants have unchanged match rates of 93.7% in 2020 to 93.7% in 2023. All DO applicants have an increased match rate from 90.7% in 2020 to 91.6% in 2023. Furthermore, more DOs have matched than before correlating with the opening of more DO schools. This data appears encouraging and has been reported by the AOA to be the best match in history [1]. This, however, does not correspond to all the surgical subspecialties. Overall, NRMP matches in surgical subspecialties have correspondingly been low for DO applicants with only three applicants successfully matching into neurosurgery, zero DO seniors matching in integrated plastic surgery, and one DO applicant matching in thoracic surgery in 2023 [2]. This contrasts with 2018 when a combined 12 DO seniors matched in neurosurgery between both the NRMP and NMS AOA match [3,4]. It has even been reported within the orthopedic literature that osteopathic applicants have much lower match rates than MD applicants [5]. Therefore, we sought to evaluate the overall trends in surgical and surgical subspecialty match rates beginning in 2020 for DO and MD applicants to identify trends and proffer solutions.

## Materials and methods

This non-funded, non-patient trial was approved by the Arrowhead Regional Medical Center institutional review board and given exempt status under protocol number 23-22. The data within the National Matching Services AOA Match in 2018 and NRMP matches from 2018 to 2023 were queried to identify trends [2-4,6-9]. The year 2020-2023 had the most comprehensive released records by NRMP. Investigated surgical subspecialties for this analysis include categorical general surgery (GS), neurosurgery (NSGY), orthopedic surgery (Ortho), vascular surgery (VS), thoracic surgery (TS), integrated plastic surgery (PS), and ENT. Chi-squared testing, student t-tests, and descriptive statistics were used to analyze data. Match rates are defined as the number of matched candidates divided by the number of total applicants for US DO Seniors and US MD Seniors. Chi-square testing was completed to identify statistically significant differences in DO and MD match rates. DO and MD match rates were calculated by taking the number of matched DO and MD senior candidates and dividing them by the number of DO and MD candidates respectively. Expected match rates in chi-squared testing were calculated by utilizing the total number of open positions each year versus the total number of DO and MD senior applicants. The public ACGME data system was queried to evaluate program director credentials for each of specialty [10]. Linear regression was used to evaluate the effects of a program director degree on the osteopathic candidate match rate. The NRMP program director survey for 2022 was also evaluated to assess impressions and trends in ranking DO applicants [11]. A p-value of < 0.05 was considered statistically significant.

## Results

NRMP match data 2020-2023 [2,6,8,9]

The 2023 match rate for MD seniors was 94.5% and for DO seniors was 93.1%. Overall, 2,847 positions were unfilled in the NRMP match, with an unfilled rate of 1.2%, which was reported to be the lowest in history. The breakdown of selected surgical subspecialties yields a stark contrast to these numbers as seen in Tables 1-3. Table 3 shows there are significantly less than expected matched DO seniors for all selected subspecialties than expected, except for thoracic surgery which may be an effect of a low sample size for applicants with only 22 total DO senior applicants in thoracic surgery from 2020 to 2023.

**Table 1:** Match Statistics for DO and MD Senior Applicants for Selected Surgical Subspecialties DO match rate is defined as the number of matched DO applicants/total DO applicants; MD match rate is defined as the number of matched MD applicants/total MD applicants. Match data appended from National Resident Matching Program match data sets [2,6,8,9]. ENT: otolaryngology; DO: osteopathic; MD: allopathic

Specialty and Year	Matched DO Applicants	Total DO Applicants	Total Positions Available	Total Filled Positions	Ratio Matched DO Available Positions	DO Mach Rate	Matched MD Applicants	Total MD Applicants	MD Match Rate	Total Applicants (MD and DO)	Proportion of Total Applicants that Were DO	Matched DO Applicants Out of Total Applicants
2023 Neurosurgery	3	12	243	240	0.0123	0.25	211	271	0.7786	373	0.0322	0.008
2022 Neurosurgery	9	24	240	240	0.0375	0.375	202	275	0.7345	391	0.0614	0.023
2021 Neurosurgery	6	14	234	234	0.0256	0.4286	198	269	0.7361	402	0.0348	0.0149
2020 Neurosurgery	3	18	232	232	0.0129	0.1667	203	273	0.7436	397	0.0453	0.0076
2023 Thoracic Surgery	1	6	49	49	0.0204	0.1667	41	95	0.4316	138	0.0435	0.0072
2022 Thoracic Surgery	1	4	47	47	0.0213	0.25	41	76	0.5395	111	0.036	0.009
2021 Thoracic Surgery	1	6	46	45	0.0217	0.1667	43	89	0.4831	129	0.0465	0.0078
2020 Thoracic Surgery	1	8	38	38	0.0263	0.125	32	78	0.4103	120	0.0667	0.0083
2023 Plastic Surgery	0	5	207	2017	0	0	191	255	0.749	332	0.0151	0
2022 Plastic Surgery	0	10	194	194	0	0	173	281	0.6157	351	0.0285	0
2021 Plastic Surgery	2	20	187	187	0.0107	0.1	167	239	0.6987	329	0.0608	0.0061
2020 Plastic Surgery	0	13	180	180	0	0	165	236	0.6992	291	0.0447	0
2023 General Surgery	243	432	1670	1667	0.1455	0.5625	1062	1466	0.7244	3100	0.1394	0.0784
2022 General Surgery	212	397	1622	1619	0.1307	0.534	1059	1467	0.7219	3071	0.1293	0.069
2021 General Surgery	228	367	1569	1564	0.1453	0.6213	1029	1405	0.7324	2908	0.1262	0.0784
2020 General Surgery	202	340	1536	1531	0.1315	0.5941	1033	1378	0.7496	2713	0.1253	0.0745
2023 Vascular Surgery	4	17	93	92	0.043	0.2353	75	92	0.8152	159	0.1069	0.0252
2022 Vascular Surgery	1	16	84	84	0.0119	0.0625	72	100	0.72	168	0.0952	0.006
2021 Vascular Surgery	3	13	79	79	0.038	0.2308	65	94	0.6915	181	0.0718	0.0166
2020 Vascular Surgery	5	10	75	73	0.0667	0.5	61	82	0.7439	153	0.0654	0.0327
2023 ENT	23	34	373	371	0.0617	0.6765	310	379	0.8179	493	0.069	0.0467
2022 ENT	21	41	361	361	0.0582	0.5122	316	463	0.6825	574	0.0714	0.0366
2021 ENT	16	37	350	350	0.0457	0.4324	310	454	0.6828	559	0.0662	0.0286
2020 ENT	17	33	350	348	0.0486	0.5152	310	421	0.7363	505	0.0653	0.0337
2023 Orthopedic Surgery	119	237	899	899	0.1324	0.5021	690	947	0.7286	1425	0.1663	0.0835
2022 Orthopedic Surgery	111	205	875	875	0.1269	0.5415	705	1086	0.6492	1470	0.1395	0.0755
2021 Orthopedic Surgery	107	173	868	866	0.1233	0.6185	699	934	0.7484	1289	0.1342	0.083
2020 Orthopedic Surgery	112	177	849	844	0.1319	0.6328	686	867	0.7912	1192	0.1485	0.094

**Table 2:** Total DO and MD Senior Applicants Applying to Summative Selected Surgical Subspecialties in NRMP p=0.000145 when comparing overall DO and MD match rates. Match data appended from National Resident Matching Program (NRMP) data sets where-in totals correlate to summative totals of applicants and matched applicants within the selected surgical subspecialties [2,6,8,9]. DO: osteopathic; MD: allopathic

Match Year	Total DO Applied (n)	Total DO Matched (n)	DO Match Rate (%)	Total MD Applied (n)	Total MD Matched (n)	MD Match Rate (%)
2023	743	393	52.8	3505	2580	73.6
2022	697	355	50.9	3748	2568	68.5
2021	630	363	57.6	3484	2511	72.1
2020	599	340	56.8	3335	2490	74.7

**Table 3:** Chi-Squared Analysis of DO Matches Versus Expected Match Rate Matched resident values appended from National Resident Matching Program data sets [2,6,8,9]. ENT: otolaryngology; DO: osteopathic; MD: allopathic

Neurosurgery		P-value		0.0000254483
	2023	2022	2021	2020
Matched DO Seniors	3	9	6	2
Expected Overall Match Rate	0.858657	0.802676	0.826855	0.797251
Expected Number of Matched DO Seniors	10.30389	19.26421	11.57597	14.35052
Thoracic Surgery		P-value		0.164816139
Matched DO Seniors	1	1	1	1
Expected Overall Match Rate	0.485149	0.5875	0.484211	0.44186
Expected Number of Matched DO Seniors	2.910891	2.35	2.905263	3.534884
Plastic Surgery		P-value		9.53918E-07
Matched DO Seniors	0	0	2	0
Expected Overall Match Rate	0.796154	0.666667	0.722008	0.722892
Expected Number of Matched DO Seniors	3.980769	6.666667	14.44015	9.39759
General Surgery		P-value		2.32567E-35
Matched DO Seniors	243	212	228	202
Expected Overall Match Rate	0.879874	0.870172	0.88544	0.894063
Expected Number of Matched DO Seniors	380.1054	345.4582	324.9565	303.9814
Vascular Surgery		P-value		1.63115E-09
Matched DO Seniors	4	1	3	5
Expected Overall Match Rate	0.853211	0.724138	0.738318	0.815217
Expected Number of Matched DO Seniors	14.50459	11.58621	9.598131	8.152174
ENT		P-value		0.010694132
Matched DO Seniors	23	21	16	17
Expected Overall Match Rate	0.903148	0.71627	0.712831	0.770925
Expected Number of Matched DO Seniors	30.70702	29.36706	26.37475	25.44053
Orthopedic Surgery		P-value		1.42602E-08
Matched DO Seniors	119	111	107	112
Expected Overall Match Rate	0.759291	0.677769	0.784101	0.813218
Expected Number of Matched DO Seniors	179.9519	138.9427	135.6495	143.9397

**Figure 1:**
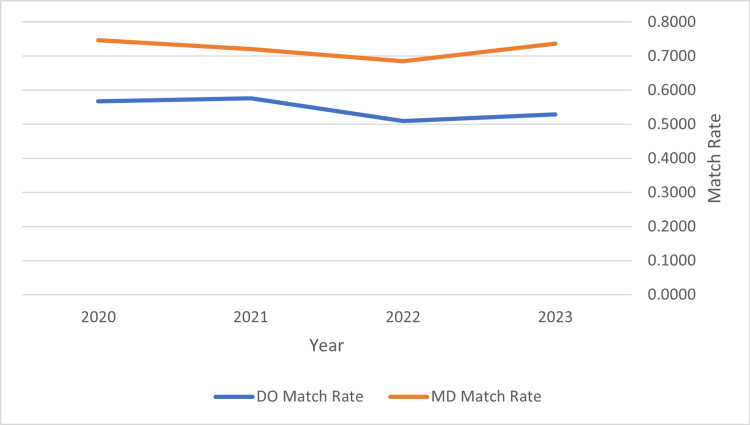
Trends in Overall Selected Surgical Subspecialty Match Rate Overall selected surgical subspecialty match rates are seen above comparing DO and MD seniors, Match rates are seen in Table 2. Data used in calculations appended from National Resident Matching Program data sets [2,6,8,9].

**Figure 2:**
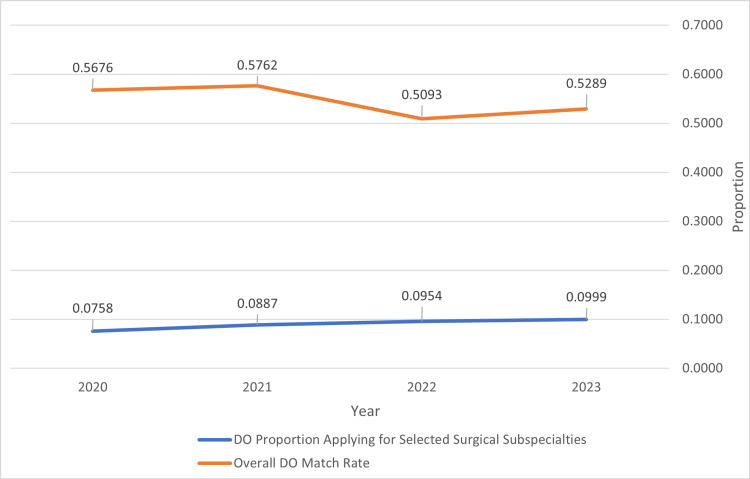
DO Proportion Applying for Specialties and Match Rate Trends An increasing proportion of DO seniors applying to these selected surgical subspecialties is seen in Figure 2 while match rates remain between 52.89% and 57.62%. Calculations were completed using match data from National Resident Matching Program data sets [2,6,8,9]. Numerical values above each line correlate the proportion of applicants applying to selected surgical subspecialties and overall match rates for DO applicants for each year. DO: osteopathic

When match rates from Table 2 were compared between specialties, it was found that the overall match was significantly different between MD and DO applicants with a p-value of 0.00145 for a two-tailed t-test. These overall specialty match rates are seen below in Figure 1. It was also identified that there is an increasing proportion of DOs applying to these surgical subspecialties with a strong linear relationship with a correlation coefficient (R^2) of 0.9441. This is demonstrated in Figure 2.

Overall match rates for the selected subspecialty surgical residencies when combining all 2020-2023 applicants are seen below in Table 4.

**Table 4:** Total Match Rates for Selected Surgical Subspecialties Match rates are calculated from reports from the National Resident Matching Program [2,6,8,9]. PS: plastic surgery, TS: thoracic surgery, VS: vascular surgery, NSGY: neurosurgery, ENT: otolaryngology, Ortho: orthopedic surgery, GS: general surgery; DO: osteopathic; MD: allopathic

Specialty Match (2020-2023)	DO Match Rate (%)	MD Match Rate (%)
PS	4.2	68.8
TS	16.7	46.5
VS	23.2	74.2
NSGY	30.9	74.9
ENT	53.1	72.6
Ortho	56.9	72.5
GS	57.6	73.2

**Figure 3:**
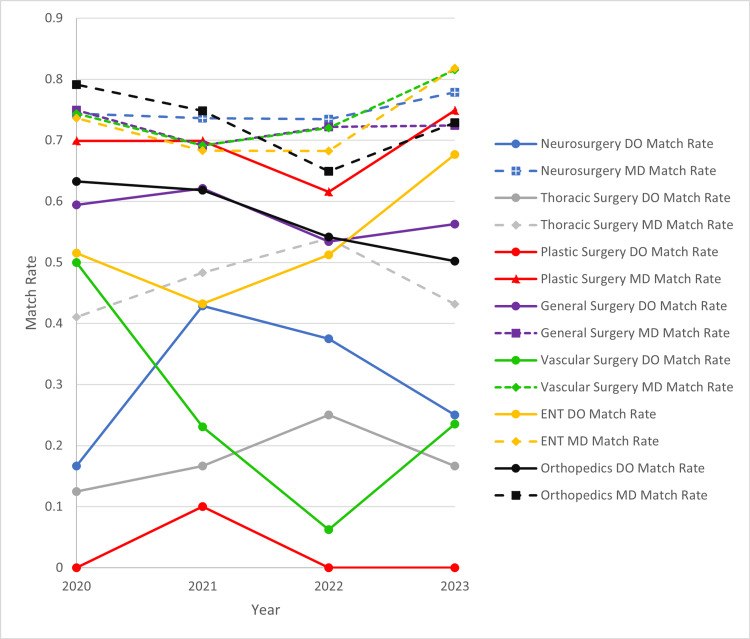
Selected Surgical Subspecialty Match Rates by Year and Specialty DO and MD selected subspecialty match rates are plotted by year. DO match rates are seen in plots with a solid line while MD match rates are plotted with segmented lines. Colors are coordinated by selected surgical subspecialty. Match rates are calculated from reports from the National Resident Matching Program [2,6,8,9]. ENT: otolaryngology; DO: osteopathic; MD: allopathic

Specific surgical subspecialty match rates are seen below in Figure 3 and in Table 5. It is noted that match rates between DO and MD applicants in all surgical subspecialties were significantly lower in DO applicants compared to MD applicants.

**Table 5:** Selected Surgical Subspecialty Match Rates by Year Match data for analysis appended from published National Resident Matching Program data sets [2,6,8,9]. ENT: otolaryngology; DO: osteopathic; MD: allopathic

	Neurosurgery		p-value
	DO match rate	MD match rate	0.000324403
2023	0.25	0.778598	
2022	0.375	0.734545	
2021	0.428571	0.736059	
2020	0.166667	0.74359	
	Thoracic Surgery		p-value
	DO match rate	MD match rate	0.00030925
2023	0.166667	0.431579	
2022	0.25	0.539474	
2021	0.166667	0.483146	
2020	0.125	0.410256	
	Plastic Surgery		p-value
	DO match rate	MD match rate	1.98041E-06
2023	0	0.74902	
2022	0	0.615658	
2021	0.1	0.698745	
2020	0	0.699153	
	General Surgery		p-value
	DO match rate	MD match rate	0.000668152
2023	0.5625	0.72442	
2022	0.534005	0.721881	
2021	0.621253	0.691489	
2020	0.594118	0.749637	
	Vascular Surgery		p-value
	DO match rate	MD match rate	0.002104564
2023	0.235294	0.815217	
2022	0.0625	0.72	
2021	0.230769	0.691489	
2020	0.5	0.743902	
	ENT		p-value
	DO match rate	MD match rate	0.017570499
2023	0.676471	0.817942	
2022	0.512195	0.682505	
2021	0.432432	0.682819	
2020	0.515152	0.736342	
	Orthopedics		p-value
	DO match rate	MD match rate	0.011203815
2023	0.50211	0.728617	
2022	0.541463	0.649171	
2021	0.618497	0.748394	
2020	0.632768	0.791234	

Match data in 2018 and 2019 are limited in evaluation given the lack of available applicant data. Available data from the 2018 and 2019 NRMP match and the 2018 AOA National Matching Services match are appended below in Tables 6-8 [3,4,7]. In 2019, the overall DO match rate for all specialties was 84.6% in the NRMP match; 6,001 applicants submitted rank lists, and 5,076 matched. In 2018, the NRMP match rate for DOs in all specialties was 81.7% overall, with 3,771 total matched of 4,617 DO applicants. In 2018, of DO medical school graduates, 4,290 graduates did not participate in the AOA match and instead participated in the NRMP match, an alternative match, or did not seek residency placement. There were only 2,833 total AOA participants in 2018 in all specialties. Of the 2,833 participants in the AOA National Matching Services match, 1,954 successfully matched with 879 not matching in AOA programs. However, of those 879, data is not available for how many participated and were successful in the NRMP match.

**Table 6:** 2019 ACGME Match Data for DO Seniors Data obtained from published National Resident Matching Program match data sets [7]. ENT: otolaryngology; DO: osteopathic; ACGME: Accreditation Council for Graduate Medical Education

Specialty	Specialty Number Matched Per Number Applied	Total Open Positions Available for Match Versus Total Filled Positions	Percent DOs Matched of Specialty Positions Available
Neurosurgery	4	232/231	1.7%
Thoracic surgery	0	37/37	0%
Plastic surgery	2	172/172	1.2%
Gen surgery	143	1432/1432	10%
Vascular surgery	5	66/64	7.6%
ENT	13	328/328	4.0%
Orthopedics	15	755/752	2%

**Table 7:** 2018 ACGME match results for DO Seniors Data appended from previously published National Resident Matching Program data sets [3]. ENT: otolaryngology; DO: osteopathic; ACGME: Accreditation Council for Graduate Medical Education

Specialty	Specialty Number Matched Per Number Applied	Total Open Positions Available for Match Versus Total Filled Positions	Percent DOs Matched of Specialty Positions Available
Neurosurgery	3	225/225	1.3%
Thoracic surgery	0	36/36	0%
Plastic surgery	2	168/167	1.2%
General surgery	83	1319/1314	6.3%
Vascular surgery	1	60/58	1.7%
ENT	3	315/303	1.0%
Orthopedics	5	742/738	0.7%

**Table 8:** 2018 National Matching Services Match Results (AOA Match) Data obtained from published National Matching Services data sets [4]. ENT: otolaryngology; AOA: American Osteopathic Association; DO: osteopathic

Specialty	Specialty Number Matched	Total Positions Available for Match Per Total Filled	Percent DOs Matched of Specialty Positions Available	Match Rate of Those DOs That Applied
Neurosurgery	9	10	90%	Unavailable
Thoracic surgery	Unavailable	Unavailable	Unavailable	Unavailable
Plastic surgery	Unavailable	Unavailable	Unavailable	Unavailable
Gen surgery	118	121	97.5%	Unavailable
Vascular surgery	Unavailable	Unavailable	Unavailable	Unavailable
ENT	19	21	90.5%	Unavailable
Orthopedics	115	116	99.1%	Unavailable

Overall, there were 268 total AOA positions in 2018 in the selected surgical specialties of neurosurgery, general surgery, ENT, and orthopedics; those positions were only available to DO applicants through the AOA National Matching Services match. When combined with the NRMP match, the total positions within the studied specialties were 3,333. In 2023, that total had a disproportionate rise to 3,535 when compared to the number of total medical school graduates.

The growth rate in surgical subspecialty-matched positions does not parallel the rate of increased numbers of DOs within the primary care specialties of family medicine, internal medicine, medicine-pediatrics combined, and pediatrics. Within these primary care specialties, the DO applicants made up 20.85% of matched applicants in 2020 and 22.5% of matched applicants in 2023. When looking at all matched applicants including the 2018 AOA match statistics, DOs who successfully matched in the investigated selected surgical subspecialties made up approximately 1.11% of matched applicants overall. This number has decreased to 1.05% in 2023.

The distribution of program directors in 2023 was also evaluated using the public ACGME data system database in regards to their degree status of DO, MD, or MBBS [10]. It was found that DO program directors make up a very small proportion of program directors within the evaluated selected surgical subspecialties. The distribution of these credentials is seen in Table 9. A regression analysis identified that there is a linear relationship between the ratio of DO program directors and match rates of DO applicants for each matched selected surgical subspecialty with an R square value of 0.75. This relationship is statistically significant with a p-value of 0.012. The NRMP program director directors survey in 2022 was queried for responses on opinions on ranking DO seniors [11]. This is tabulated below in Table 10.

**Table 9:** Credential Type of All Program Directors in Each Surgical Specialty Credentials are obtained through the public Accreditation Council for Graduate Medical Education accreditation data systems search [10]. PS: plastic surgery; TS: thoracic surgery; VS: vascular surgery; NSGY: neurosurgery; ENT: otolaryngology; Ortho: orthopedic surgery; GS: general surgery; DO: osteopathic; MD: allopathic

Specialty	Number of Programs	Number of DO Program Directors	Number of MBBS Program Directors	Number of MD Program Directors	Proportion of Program Directors That Are DOs (%)
NSGY	117	2	0	115	1.7
TS	34	1	0	33	2.9
PS	88	0	1	87	0.0
GS	356	35	0	321	9.8
VS	75	0	1	74	0.0
ENT	131	13	0	118	9.9
Ortho	208	28	0	180	13.5

**Table 10:** 2022 Program Director Survey Responses on Ranking DO Applicants Data were obtained from the public National Resident Matching Program program director survey data set [11]. ENT: otolaryngology; DO: osteopathic

Specialty	Returned Surveys (N)	Seldom Rank DO Senior (%)	Never Rank DO Senior (%)	Seldom or Never Rank DO Senior (%)
Neurosurgery	28	33	56	89
Thoracic Surgery	Unavailable	Unavailable	Unavailable	Unavailable
Plastic Surgery	17	50	25	75
General Surgery	103	33	13	46
Vascular Surgery	10	44	22	66
ENT	39	48	33	81
Orthopedics	52	39	22	61

## Discussion

The AOA and ACGME match merger has opened doors for DO students and residents to seek opportunities that may have been largely unavailable prior to the AOA and ACGME merger. Evaluating the match data, it has been found that DOs have significantly lower match rates compared with MD applicants in NSGY, TS, GS, VS, PS, ENT, Ortho, and overall, within the summative selective surgical subspecialties. These surgical subspecialty match rate concerns were initially brought up by Etheart in 2021, however, it appears little has changed over the last two years [12]. Additionally, there was concern in 2021 that the resultant loss of AOA surgical programs with the increase in additional DO schools would result in increased competition, adversely affecting the opportunity for surgical training for graduating DOs [13]. Within the USA, there are additional concerns about a growing physician shortage driven by an aging workforce and burnout rates [14]. This shortage is predicted to involve all specialties with shortage totals between 37,800 and 124,000 physicians by 2034. It is estimated that the shortage is 15,800 to 30,200 within surgical subspecialties [15]. Significantly fewer than expected DOs match in all the investigated surgical subspecialties except in TS for which the total number of DO applicants may limit interpretation, with only 22 applicants from 2020 to 2023. Table 10 also identifies the large number of program directors who have identified that they seldom or never rank DO applicants [11] and the need to promote the unique qualifications of osteopathic applicants. This issue is present beyond these selected surgical subspecialties with 8% of surveyed program directors in all specialties stating they never rank a US DO senior and 20% “seldom” rank a DO senior in 2022 [11]. However, this same program director survey sought from those that said they did a holistic review of the candidate the discrete factors of the specific holistic review. Although 88% looked at the applicant's personal attributes; 85% applicant's interests; 81% applicant's interpersonal skills, ethics, and professionalism; 81% applicant's personal experiences; and 54% applicant's geographical preferences, there was no mention of the applicants’ medical philosophy of holistic patient-centered care, or the integration of the mind, body, and spirit. It is therefore important to promote increasing education and training of future surgical subspecialists from all backgrounds and training environments, to ensure a more comprehensive and diverse working cohort of residents to best address the individual patient’s needs. This education of program directors, faculty, department chairs, and practice administrators need to stress the strengths of the distinctive osteopathic medical students and the osteopathic philosophy acquired and ingrained during medical school, which aligns with the modern and desired medical model of comprehensive patient care.

The history of the osteopathic medical education structure was community-based and different from the allopathic structure. It stemmed from the distinct business models of osteopathic and allopathic medical schools, which was in part due to the difference in the accreditation standards, which was in part due to philosophy. The difference is a contributory factor to the disparity between DO and MD medical student applicants successfully matching into competitive surgical and subspecialty residencies. Many osteopathic medical schools, long prior to the single accreditation system, relied on basic medical science faculty, primary care clinical faculty, certified osteopathic manipulative medicine physicians, and a few consulting specialists and subspecialists. This patient-centered faculty family provided a comprehensive osteopathic curriculum during the first two years of didactic medical school instruction, with the philosophy molded by the mind-body-spirit approach of the primary care and specialist physicians to provide strong hands-on instruction, examination, and holistic care of the patient. In the past, osteopathic medical schools had no distinct departments and department chairs in many of the specialty disciplines; this has changed for some time. The third and fourth-year osteopathic medical school curricula were centered on clinical clerkships in primary care and the required specialty services. During this time, there was the credentialing of specific providers for the required clinical clerkships but there were few dedicated clinical faculty providing oversight of each specific discipline, and virtually none for the surgical and subspecialties. The professional oversight of the clinical provider, training efficacy, and evaluation and assessment of student progress was required to be performed by the clinical dean of clinical rotations.

When the AOA and the American Association of Colleges of Osteopathic Medicine (AACOM) agreed to transition the AOA-certified residencies by 2020 into the single ACGME accreditation, it resulted in the need for the osteopathic medical schools to complete the transition of their business model. This required the osteopathic medical school to obtain dedicated specialty faculty to provide curriculum input during all four years of school. This strengthened the integration of the osteopathic philosophy within the didactics provided by both primary and specialty care osteopathic physicians. This increased involvement of the specialty physician in the osteopathic medical school structure better aligned with that seen in the allopathic medical schools which was needed to prepare all students for their choice of career. This change has become essential for the osteopathic medical student to be considered in any ACGME residency as there must be a letter of support from the department chair of that specific discipline, especially in the highly competitive surgical subspecialty programs. In addition, since osteopathic medical schools have not previously dedicated clinical faculty in the subspecialty disciplines, there has been a growing but a paucity of subspecialty research opportunities for these medical students, which is also an important consideration in ranking a competitive candidate during the residency application process.

To address these disparities and improve the equity of these diverse candidates being selected for surgical and surgical subspecialty residencies, the following 12 steps are suggested.

Develop an office of advocacy

Since the SAS, there has been a new diversity in medical residency philosophy with much-needed education about the equality and inclusivity of applicants. This education should be focused on residency program directors, faculty, department chairs, and practice administrators. The AOA along with other specialty organizations can identify issues affecting DO medical students' successful matching into residencies, residents into fellowships, and graduates into practice. The AOA and its affiliates can then use its resources to address the potential biases discussed above. The AOA can also provide information about the benefits of including DO medical students with their holistic philosophy and patient-centered approach in residencies and improve equality of education. The DO family can add information about the qualifications of DO graduates, DO quality of care, the public cries for caring for the whole patient and patient unit, and the satisfaction of the healing touch for each patient. The advocacy should then come from the many associated AOA organizations across local, state, regional, and national organizations, bringing the family of medicine together to make it stronger.

Build DO membership in osteopathic organizations

For there to be significant gains for DO medical students successfully matching into surgical subspecialty residencies, there need to be viable DO organizations. Organizations are viable when there is a strong membership, and the members are active. DO societies need to offer many reasons for membership, including advocacy and the continuation of the profession. Advocacy is the primary means to continue promoting the value of the holistic osteopathic profession in any specialty and in medicine. Without advocacy, the concerns of osteopathic physicians would largely go unheard, and DO physicians would have to rely on other organizations that do not fully comprehend the challenges of the inherent bias. Furthermore, osteopathic organizations at the local, state, and national levels can work collaboratively and create opportunities for students to establish thriving mentor relationships that can lead to successful careers within all specialties and lead to higher success rates within the match for each specialty.

Promote DOs within surgical leadership

The analysis above in Table 9 identified that there are few DO program directors within these selected surgical subspecialties including zero in PS and VS. Correspondingly, no DO seniors matched in plastic surgery. There is a statistically significant relationship between DOs matching in these subspecialties to the ratio of DO program directors. The neurosurgery program director survey as referenced in Table 10 demonstrates that of those neurosurgical program directors that returned the survey, 11% were often interviewed, and 11% also often ranked DO seniors compared to 100% of MD seniors that often get interviewed and 100% that often get ranked [11]. There are no reasons given in the survey for this dichotomy. Additionally, DOs hold many leadership positions in medicine already. As such, it should be the norm for a DO to also hold leadership positions within residency programs and graduate medical education (GME). Therefore, it is prudent to advocate for increased DO leadership as program directors and within GME to try to increase osteopathic philosophy and representation within these selected fields. Lastly, intentional efforts in recruiting and retaining trainees who are interested in academics can support the growth of GME program leadership in the future.

Promote DO and MD residents to take AOA boards

The ACGME Common Program Requirements state that the qualifications of the program director must include current certification in the specialty for which they are the program director by the American Board of Medical Specialties (ABMS) or by the AOA certifying board [16]. The program director should encourage all eligible program graduates to take the certifying examination offered by the applicable ABMS member board or AOA certifying board. Furthermore, physician faculty members must have current certification in the specialty by ABMS or AOA certifying board. However, the number of applicants sitting for the AOA certifying boards in the selected surgical subspecialties has decreased. It is more likely than not that a DO program director, certified by the AOA certifying boards, would educate, permit, and even encourage qualified residents to sit for the AOA boards as they would have a better understanding of the differences in the certifying boards. Prior to SAS, only DO residents who graduated from AOA-approved programs have sat for the AOA certifying boards. As of 2022, MDs are qualified and encouraged to do so. Having additional DO and MD residents sit for the AOA certifying boards would promote AOA certifying boards as equivalent, ensure continued availability of this board certification option, and embolden programs to hire more DO board-certified program directors and faculty. To develop additional AOA certifying board diplomats, the boards must be seen as at least equivalent in quality. Ensuring that the AOA certifying boards are an accessible, less cumbersome, and desirable board certification process will allow for exceptional candidates from all training backgrounds to apply for AOA board certification and lead to increased candidate usage and promotion by program directors.

Process changes within medical schools to promote successful matching in selected surgical subspecialties

There are specific processes that need to address how osteopathic medical schools can significantly improve match rates for candidates in selected surgical subspecialties. It is noteworthy that in 2018, 98.75% of DO graduates that were seeking placement for a GME position were successful [17]. This is a value that has not yet been seen in the NRMP match. Medical schools need to interface with students directly about their needs and concerns with matching into their specialty of choice, specifically within these surgical subspecialties. DO medical schools should also consider assuring alignment in resources needed to be successful in matching into surgical specialties (accurate and meaningful advising, research, leadership opportunities, etc.). In addition, medical schools should have an annual review of their match successes to continue to modify their match strategies. There is further opportunity for these medical schools to partner with osteopathic professional societies to help promote student success in matching.

Increase institutional involvement at the osteopathic medical school level in research in surgical subspecialties

There have been numerous studies looking at factors related to successful matches within these competitive subspecialties [17-20]. In evaluating these studies several factors were noted to be critical to improve a candidate’s success in the match. These include factors related to an individual’s institution, as well as the candidate’s own individual involvement. Successful graduates tend to come from institutions with high levels of research and funded research, strong mentorship programs, dedicated interest groups in the associated specialties, schools with early subspecialty exposure for medical students, and dedicated subspecialty faculty in the associated specialty [18-21]. By increasing the amount of research conducted at a medical institution, more grant money could be obtained from national research organizations to further support the expansion of such opportunities. If it is cost-restrictive to increase funding for research, medical schools should be encouraged to seek out partnerships with local institutions that conduct research or quality improvement and connect their students to these organizations. Medical schools would not have an increase in their own financial burden when using this route. Other considerations improved through osteopathic medical schools obtaining high-profile federal and specialty grants include addressing the potential bias in considering a DO candidate for subspecialty residency positions.

Support needs for medical students to make successful matches

In addition to increasing the research at the level of the osteopathic medical school, schools may need to consider the needs of the individual medical students matching into these selected surgical subspecialties. When specifically looking at the successful match candidate in surgical subspecialties, it is important to evaluate the importance of research and specifically funded research that the applicant was involved in [18,19]. Overall, research has been found to be correlated with increased match rates in MD candidates and it has been found that MD candidates' research experiences in matched and unmatched cohorts are significantly greater than DO candidates [18]. Matched and unmatched DO candidates and MD candidates also had significantly different levels of research experience in matched and unmatched cohorts and it has been proposed that increasing scholarly activity among DO medical students may make them more competitive candidates overall [18]. When specifically looking at the surgical subspecialty of neurosurgery, it has been found that schools that have higher levels of funded research and research experience match significantly more students into neurosurgery [19]. Therefore, it is proposed that medical schools and osteopathic medical schools may need to sponsor each student to participate in research and quality improvement more heavily and invest in creating more opportunities and diversity for funded research for their students to increase their competitiveness.

Increase osteopathic medical school advocacy of medical training and board scores

There is a notable potential bias within medical education claiming that MD candidates receive training that is superior to DO candidates [22]. Contrary to that belief, within specific surgical education, DO and MD residents have been shown to have equivalent educational metrics. In fact, a recent study, investigating orthopedic surgery in training scores in all ACGME programs in 2019 (N=3,797) found that DO PGY-1 residents outperformed MD residents with scores similar to PGY 2-4s [22]. The authors concluded that DO and MD orthopedic surgery residents perform similarly on the orthopedic in-training exam PGY2-4 with equivalency in orthopedic knowledge [22]. Although not a surgical subspecialty, analysis of residents in physical medicine and rehabilitation showed that performance on the initial certifying examination has been equivalent in DO and MD applicants among board-eligible physical medicine and rehabilitation physicians [23].

Additionally, GME program leadership in surgical subspecialties requires USMLE Steps 1 and 2 for consideration in many programs with a large portion not even considering the Comprehensive Osteopathic Medical Licensing Examination (COMLEX) [11]. Although the background of this requirement has not been fully investigated, it could be attributed to a lack of familiarity with COMLEX Levels 1 and 2 scores. To improve equity in opportunity and decrease discrimination, ACGME could create Core Requirements for faculty development surrounding education and understanding of COMLEX testing and scoring for DO applicants. This would save DO medical students time, money, and the additional stress of taking two certifying examinations when only one is required to become licensed in any US state.

Mentorship and osteopathic surgical organizations

The importance of mentorship cannot be underestimated regarding successful match rates for candidates in all residency specialties, let alone surgical subspecialty education. Mentors can provide valuable advice, guidance, and leadership for candidates early in training to help them navigate the process of applications and interviewing. They can also allow the candidate to better understand the position they are applying for and develop strong long-lasting relationships. Mentors are also able to connect students to various resources that will help improve candidacy from the first day of medical school for a successful match and direct interested candidates towards ways they can be successful. These mentors can help mold these students as early as the first year of medical school, identify potentially successful candidates, teach them nuances of the specialty, and provide letters of recommendation. These mentors are proven to significantly improve a candidate’s ability to match. Specifically, within VS it has been found that medical students with strong mentorship prior to applying to residency have significantly increased rates of matching [21]. Residency fairs can also provide venues where surgical organizations can educate first-year students about the many career choices. Osteopathic surgical subspecialty physicians should be utilized to present talks and lectures to DO medical students. The self-selected students can then use the resources of the surgical organizations to identify interested and active mentors to further interact and guide their interests.

Medical school surgical subspecialty interest groups

Interest groups have also been found to be vital for a medical student’s success in matching these selected surgical subspecialties. A recent evaluation of successfully matched neurosurgery residents and the schools they graduated from, identified that graduates who apply from schools with specific interest groups for neurosurgery have higher success rates in matching [19]. These reasons include increased evidence of interest from an earlier stage of training, more time to complete related research projects, and then ultimately, the development of strong mentor relationships. In addition, surgical subspecialty interest groups increase early exposure to medical students. They can also have outreach to premedical students, strengthening a pipeline to these specialties via osteopathic education.

Early medical school exposure to surgical subspecialties

Early exposure to surgical subspecialties has similarly been identified to be related to improved match rates. When VS and NSGY were evaluated, applicants who had earlier exposure in medical school e.g., years one and two, had improved match rates [19,21]. These candidates are thought to develop a strong understanding of their subspecialties and more applicants can identify a potential career trajectory from an earlier phase. This earlier exposure may lead to attracting more osteopathic applicants who then become better candidates through their relationships with their surgical mentors. Early exposure to these selected surgical subspecialties through the first year of medical school may lead to stronger applicants applying and matching their desired specialties. Furthermore, an early and formal rotation in preclinical years on a teaching service can help students understand the training process and day-to-day workload of physicians and trainees in these surgical subspecialties.

Medical school faculty from surgical subspecialties

Additional areas for improvement come within the intrinsic structuring of departments. It has been found that within these selected surgical subspecialties having specific subspecialty members on medical school faculty improves match rates for candidates applying from their institutions [19]. This was studied within NSGY where it was found that schools with higher numbers of NSGY faculty in their medical schools have better match rates in neurosurgery [19]. This is theorized to be due to the increased early exposure, research projects, mentorship, and education within that surgical subspecialty allowing for improved success in audition rotations, interviews, and for candidates creating successful applications. A direct benefit would be creating a Surgical Chair position at every osteopathic medical school to be the liaison for students pursuing surgical training. This Chair would be able to help students make connections to surgeons in their chosen field to fortify the previously discussed aspects of ensuring a successful match.

## Conclusions

Within selected surgical subspecialties of GS, NSGY, TS, VS, ENT, and PS match rates remain significantly lower for DO applicants than MD applicants. Furthermore, applications to these selected surgical specialties are rising within the DO applicant pool but match rates continue to remain low or even fall. Systemic intentional efforts are needed in to achieve equity in the opportunity for DO students to train in surgical subspecialties. National osteopathic and allopathic organizations and osteopathic medical schools must increase efforts to promote success for these osteopathic candidates in the match by improving mentorship, research, and early surgical subspecialty exposure. Advocacy for osteopathic applicants should be evaluated and strengthened to address the unsubstantiated stigmas against DO applicants and board certification. Furthermore, the osteopathic philosophy of patient-centered holistic care must be promoted as one of the reasons to give the matching osteopathic applicants a holistic review. The osteopathic family must strengthen all its organizations and must promote more successful residency, fellowship, and practice applications. There must be the creation of dedicated offices directed at advocating for the inclusion of these diverse osteopathic applicants to promote success in their selected fields, to strengthen medicine, and to fill the physician shortages of the future.
